# *catena*-Poly[[aqua­(5,5′-dimethyl-2,2′-bipyridine-κ^2^ *N*,*N*′)copper(II)]-μ-2,2′-oxydibenzoato-κ^2^ *O*:*O*′]

**DOI:** 10.1107/S160053681002622X

**Published:** 2010-07-10

**Authors:** Chong-Zhen Mei, Han-Lin Xiong, Peng Zhang

**Affiliations:** a Institute of Environmental and Municipal Engineering, North China University of Water Conservancy and Electric Power, Zhengzhou 450011, People’s Republic of China

## Abstract

In the title compound, [Cu(C_14_H_8_O_5_)(C_12_H_12_N_2_)(H_2_O)]_*n*_, the Cu^II^ ion is penta­coordinated in a square-pyramidal geometry. Two N atoms of the chelating 5,5′-dimethyl-2,2′-bipyridine (dbp) ligand and two O atoms of two different 2,2′-oxydibenzoic (odb) ligands occupy the basal plane while the water O atom completes the square-pyramidal geometry at the apical site. The non-water N_2_O_2_ donor atoms are nearly coplanar, with a mean deviation from the least-squares plane of 0.0518 (11) Å and the Cu atom is displaced by 0.1507 (11) Å from this plane towards the apical water O atom. Further coordination *via* the 2,2′-oxydibenzoate anions forms a one-dimensional coordination polymer extending parallel to [010]. In the crystal structure, O—H⋯O hydrogen bonds link the mol­ecules into a two-dimensional supra­molecular structure.

## Related literature

For background to the network topologies and applications of coordination polymers, see: Yaghi *et al.* (1998 ▶). For structures containing odb ligands, see: Gong *et al.* (2009 ▶); Hong (2008*a* ▶,*b* ▶); Wang *et al.* (2010 ▶); Yu (2008 ▶); Xu *et al.* (2008*a* ▶,*b* ▶). For complexes with 5,5′-dimethyl-2,2′-bipyridine (dbp), see: Zhao & Bai (2009 ▶); Khalighi *et al.* (2008 ▶); Kalateh *et al.* (2008 ▶); Dong *et al.* (2009 ▶); Ahmadi *et al.* (2008 ▶, 2010 ▶).
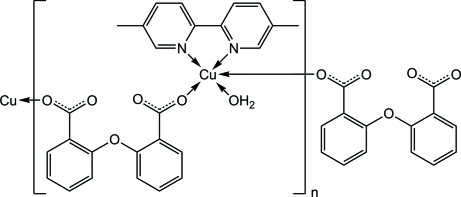


## Experimental

### 

#### Crystal data

[Cu(C_14_H_8_O_5_)(C_12_H_12_N_2_)(H_2_O)]
*M* *_r_* = 522.00
Monoclinic, 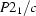

*a* = 7.4235 (11) Å
*b* = 17.475 (3) Å
*c* = 18.053 (3) Å
β = 98.188 (3)°
*V* = 2318.0 (6) Å^3^
*Z* = 4
Mo *K*α radiation
μ = 0.99 mm^−1^
*T* = 296 K
0.20 × 0.18 × 0.16 mm

#### Data collection

Bruker SMART APEXII CCD area-detector diffractometer
Absorption correction: multi-scan (*SADABS*; Bruker, 2009 ▶) *T*_min_ = 0.827, *T*_max_ = 0.858
12080 measured reflections
4071 independent reflections
3150 reflections with *I* > 2σ(*I*)
*R*_int_ = 0.042

#### Refinement

*R*[*F* ^2^ > 2σ(*F* ^2^)] = 0.043
*wR*(*F* ^2^) = 0.097
*S* = 1.04
4071 reflections
318 parameters
H-atom parameters constrained
Δρ_max_ = 0.32 e Å^−3^
Δρ_min_ = −0.22 e Å^−3^

### 

Data collection: *APEX2* (Bruker, 2009 ▶); cell refinement: *SAINT* (Bruker, 2009 ▶); data reduction: *SAINT*; program(s) used to solve structure: *SHELXTL* (Sheldrick, 2008 ▶); program(s) used to refine structure: *SHELXTL*; molecular graphics: *DIAMOND* (Brandenburg, 2010 ▶); software used to prepare material for publication: *SHELXTL*.

## Supplementary Material

Crystal structure: contains datablocks I, global. DOI: 10.1107/S160053681002622X/nk2034sup1.cif

Structure factors: contains datablocks I. DOI: 10.1107/S160053681002622X/nk2034Isup2.hkl

Additional supplementary materials:  crystallographic information; 3D view; checkCIF report

## Figures and Tables

**Table:** 

Cu1—O5	1.915 (2)
Cu1—O2^i^	1.9370 (19)
Cu1—N2	2.000 (2)
Cu1—N1	2.018 (2)
Cu1—O1*W*	2.388 (2)
O2—Cu1^ii^	1.9371 (19)

**Table:** 

O5—Cu1—O2^i^	95.15 (8)
O5—Cu1—N2	165.19 (9)
O2^i^—Cu1—N2	93.03 (9)
O5—Cu1—N1	90.14 (10)
O2^i^—Cu1—N1	171.74 (10)
N2—Cu1—N1	80.48 (10)
O5—Cu1—O1*W*	96.74 (8)
O2^i^—Cu1—O1*W*	93.83 (8)
N2—Cu1—O1*W*	95.01 (8)
N1—Cu1—O1*W*	91.83 (9)

Symmetry codes: (i) 
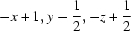
; (ii) 
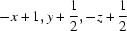
.

**Table 2:** Hydrogen-bond geometry (Å, °)

*D*—H⋯*A*	*D*—H	H⋯*A*	*D*⋯*A*	*D*—H⋯*A*
O1*W*—H1*WA*⋯O4	0.85	1.99	2.750 (3)	148
O1*W*—H1*WB*⋯O3^iii^	0.85	2.13	2.972 (3)	171

Symmetry code: (iii) 
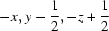
.

**Fig. 1.**
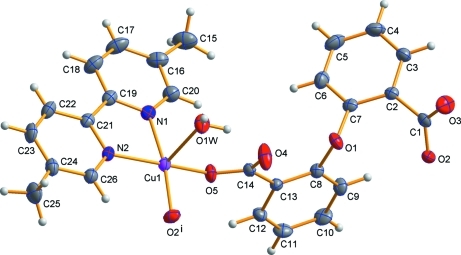
The molecular structure of the title compound with the atom-labelling scheme. Displacement ellipsoids are drawn at the 30% probability level. H atoms are presented as small spheres of arbitrary radius. [Symmetry code: (i) -x+1, y-1/2, -z+1/2]

**Fig. 2.**
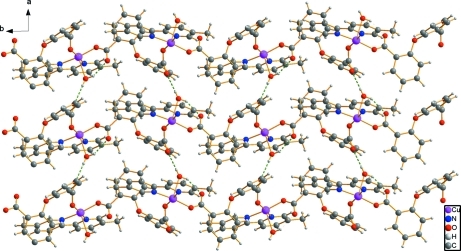
A view of the structure along the c axis. dashed lines indicate the hydrogen-bonding.

## supplementary crystallographic information

### Comment

5,5'-Dimethyl-2,2'-bipyridine (dbp), is a good bidentate ligand, and numerous complexes with dbp have been prepared, such as that of Zn (Zhao & Bai, 2009; Khalighi *et al.* 2008), In (Kalateh *et al.* 2008), Cu (Dong *et al.* 2009) and Cd (Ahmadi *et al.* 2008, 2010).

Recently, great interest has been focused on the design and synthesis of coordination polymers because of their intriguing network topologies and promising applications (Yaghi *et al.* 1998). Hence we have employed 2,2'-oxydibenzoic (odb) and dbp as ligands in this work.

In the title complex the Cu^2+^ ion is is pentacoordinated, with two N atoms of chelating 5,5'-dimethyl-2,2'-bipyridine (dbp) ligand and two O atoms of two different odb ligands in the basal plane and the O atom of water molecule completing the square-pyramidal geometry from the apical site (Fig. 1). The atoms N1, N2, O5 and O2^i^ [Symmetry code: (i) -*x*+1, *y*-1/2, -*z*+1/2] are nearly coplanar, with a mean deviation from the least-squares plane of 0.0518 (11) Å, and the Cu atom is displaced by 0.1507 (11) Å from this plane towards the apical O atom. Further coordination *via* the 2,2'-oxydibenzoate anions forms a one-dimensional coordination polymer extending parallel to [010]. In the crystal structure, O—H···O hydrogen bonds link the molecules into a 2D supramolecular structure as shown in Fig. 2.

### Experimental

Copper(II) acetate dihydrate (0.5 mmol), 5,5'-dimethyl-2,2'-bipyridine (0.5 mmol) and 2,2'-oxydibenzoic acid (0.5 mmol) were placed in a 30 ml teflon-lined, stainless-steel Parr autoclave together with water (20 ml). The autoclave was heated at 393 K for a week and was subsequently cooled slowly to room temperature. Blue single crystals were obtained.

### Refinement

The approximate positions of the water H atoms, obtained from a difference Fourier map, were restrained to ideal water geometry and fixed in the final stages of refinement (O—H 0.85 Å). All other H atoms were included in calculated positions, with C—H bond lengths fixed at 0.93Å (aryl group), 0.96 Å (methyl CH_3_) and were refined in the riding-model approximation. *U*_iso_(H) values were calculated at 1.5 *U*_eq_(C) for methyl H atoms and 1.2 *U*_eq_(C, O) for the other H atoms.

### Crystal data

**Table:**

[Cu(C_14_H_8_O_5_)(C_12_H_12_N_2_)(H_2_O)]	*F*(000) = 1076
*M**_r_* = 522.00	*D*_x_ = 1.496 Mg m^−^^3^
Monoclinic, *P*2_1_/*c*	Mo *K*α radiation, λ = 0.71073 Å
Hall symbol: -P 2ybc	Cell parameters from 2235 reflections
*a* = 7.4235 (11) Å	θ = 2.1–25.6°
*b* = 17.475 (3) Å	µ = 0.99 mm^−^^1^
*c* = 18.053 (3) Å	*T* = 296 K
β = 98.188 (3)°	Block, blue
*V* = 2318.0 (6) Å^3^	0.20 × 0.18 × 0.16 mm
*Z* = 4	

### Data collection

**Table:**

Bruker SMART APEXII CCD area-detector diffractometer	4071 independent reflections
Radiation source: fine-focus sealed tube	3150 reflections with *I* > 2σ(*I*)
graphite	*R*_int_ = 0.042
ω scans	θ_max_ = 25.0°, θ_min_ = 2.3°
Absorption correction: multi-scan (*SADABS*; Bruker, 2009)	*h* = −8→8
*T*_min_ = 0.827, *T*_max_ = 0.858	*k* = −20→10
12080 measured reflections	*l* = −21→21

### Refinement

**Table:**

Refinement on *F*^2^	Primary atom site location: structure-invariant direct methods
Least-squares matrix: full	Secondary atom site location: difference Fourier map
*R*[*F*^2^ > 2σ(*F*^2^)] = 0.043	Hydrogen site location: inferred from neighbouring sites
*wR*(*F*^2^) = 0.097	H-atom parameters constrained
*S* = 1.04	*w* = 1/[σ^2^(*F*_o_^2^) + (0.050*P*)^2^ + 0.0178*P*] where *P* = (*F*_o_^2^ + 2*F*_c_^2^)/3
4071 reflections	(Δ/σ)_max_ = 0.001
318 parameters	Δρ_max_ = 0.32 e Å^−^^3^
0 restraints	Δρ_min_ = −0.22 e Å^−^^3^

### Special details

**Table:**

Geometry. All esds (except the esd in the dihedral angle between two l.s. planes) are estimated using the full covariance matrix. The cell esds are taken into account individually in the estimation of esds in distances, angles and torsion angles; correlations between esds in cell parameters are only used when they are defined by crystal symmetry. An approximate (isotropic) treatment of cell esds is used for estimating esds involving l.s. planes.
Refinement. Refinement of *F*^2^ against ALL reflections. The weighted *R*-factor wR and goodness of fit *S* are based on *F*^2^, conventional *R*-factors *R* are based on *F*, with *F* set to zero for negative *F*^2^. The threshold expression of *F*^2^ > σ(*F*^2^) is used only for calculating *R*-factors(gt) etc. and is not relevant to the choice of reflections for refinement. *R*-factors based on *F*^2^ are statistically about twice as large as those based on *F*, and *R*- factors based on ALL data will be even larger.

### Fractional atomic coordinates and isotropic or equivalent isotropic displacement parameters (Å^2^)

**Table:**

	*x*	*y*	*z*	*U*_iso_*/*U*_eq_	
Cu1	0.41788 (4)	0.62384 (2)	0.109714 (18)	0.03306 (13)	
N1	0.3306 (3)	0.65433 (15)	0.00279 (13)	0.0377 (6)	
N2	0.3278 (3)	0.52235 (13)	0.06863 (12)	0.0323 (5)	
O1	0.4265 (2)	0.93932 (11)	0.19835 (11)	0.0386 (5)	
O2	0.4863 (3)	1.08114 (12)	0.29366 (10)	0.0418 (5)	
O3	0.1882 (3)	1.09819 (15)	0.29298 (12)	0.0577 (6)	
O4	0.3634 (3)	0.77983 (13)	0.19838 (16)	0.0715 (8)	
O5	0.5373 (3)	0.72067 (11)	0.12721 (11)	0.0426 (5)	
O1W	0.1358 (3)	0.66083 (13)	0.14824 (12)	0.0539 (6)	
H1WA	0.1690	0.7023	0.1708	0.065*	
H1WB	0.0485	0.6448	0.1698	0.065*	
C1	0.3209 (4)	1.08195 (16)	0.26283 (15)	0.0336 (7)	
C2	0.2924 (3)	1.06403 (16)	0.18006 (15)	0.0299 (6)	
C3	0.1997 (4)	1.11672 (18)	0.13170 (17)	0.0416 (7)	
H3	0.1522	1.1605	0.1510	0.050*	
C4	0.1763 (5)	1.1054 (2)	0.05493 (19)	0.0591 (10)	
H4	0.1161	1.1419	0.0230	0.071*	
C5	0.2424 (5)	1.0401 (3)	0.02647 (19)	0.0661 (11)	
H5	0.2284	1.0327	−0.0250	0.079*	
C6	0.3294 (4)	0.9855 (2)	0.07367 (19)	0.0532 (9)	
H6	0.3713	0.9407	0.0542	0.064*	
C7	0.3543 (3)	0.99754 (17)	0.15049 (16)	0.0332 (7)	
C8	0.6009 (4)	0.91544 (17)	0.18894 (15)	0.0319 (6)	
C9	0.7378 (4)	0.97019 (18)	0.19715 (17)	0.0424 (8)	
H9	0.7110	1.0207	0.2076	0.051*	
C10	0.9132 (4)	0.9495 (2)	0.18986 (19)	0.0504 (9)	
H10	1.0052	0.9860	0.1957	0.060*	
C11	0.9531 (4)	0.8748 (2)	0.17389 (18)	0.0500 (9)	
H11	1.0716	0.8609	0.1687	0.060*	
C12	0.8167 (4)	0.82106 (18)	0.16569 (16)	0.0396 (7)	
H12	0.8445	0.7708	0.1546	0.048*	
C13	0.6376 (3)	0.83974 (17)	0.17359 (14)	0.0299 (6)	
C14	0.4984 (4)	0.77630 (16)	0.16651 (16)	0.0327 (7)	
C15	0.3015 (5)	0.8219 (2)	−0.1299 (2)	0.0723 (12)	
H15A	0.3508	0.8548	−0.0894	0.108*	
H15B	0.1830	0.8399	−0.1509	0.108*	
H15C	0.3802	0.8222	−0.1677	0.108*	
C16	0.2864 (4)	0.7414 (2)	−0.10098 (19)	0.0529 (9)	
C17	0.2245 (5)	0.6804 (3)	−0.14685 (19)	0.0643 (11)	
H17	0.1899	0.6886	−0.1978	0.077*	
C18	0.2136 (4)	0.6083 (2)	−0.11798 (18)	0.0552 (9)	
H18	0.1712	0.5678	−0.1491	0.066*	
C19	0.2660 (4)	0.59610 (19)	−0.04244 (16)	0.0381 (7)	
C20	0.3397 (4)	0.7244 (2)	−0.02617 (18)	0.0474 (8)	
H20	0.3842	0.7639	0.0058	0.057*	
C21	0.2604 (4)	0.52224 (18)	−0.00508 (16)	0.0351 (7)	
C22	0.1926 (4)	0.4554 (2)	−0.03889 (18)	0.0482 (9)	
H22	0.1471	0.4551	−0.0896	0.058*	
C23	0.1919 (4)	0.38961 (19)	0.0019 (2)	0.0527 (9)	
H23	0.1453	0.3448	−0.0212	0.063*	
C24	0.2601 (4)	0.38895 (18)	0.07754 (19)	0.0452 (8)	
C25	0.2616 (5)	0.3185 (2)	0.1245 (2)	0.0670 (11)	
H25A	0.3677	0.3187	0.1617	0.100*	
H25B	0.2637	0.2740	0.0933	0.100*	
H25C	0.1544	0.3174	0.1486	0.100*	
C26	0.3266 (4)	0.45785 (18)	0.10777 (17)	0.0404 (7)	
H26	0.3733	0.4592	0.1584	0.048*	

### Atomic displacement parameters (Å^2^)

**Table:**

	*U*^11^	*U*^22^	*U*^33^	*U*^12^	*U*^13^	*U*^23^
Cu1	0.0383 (2)	0.0272 (2)	0.0334 (2)	−0.00240 (16)	0.00395 (14)	−0.00399 (17)
N1	0.0390 (14)	0.0389 (15)	0.0359 (14)	−0.0015 (12)	0.0077 (11)	0.0017 (12)
N2	0.0340 (12)	0.0318 (14)	0.0316 (13)	−0.0041 (11)	0.0064 (10)	−0.0061 (11)
O1	0.0331 (11)	0.0267 (11)	0.0592 (13)	0.0026 (9)	0.0180 (9)	0.0021 (10)
O2	0.0402 (12)	0.0403 (13)	0.0417 (12)	0.0130 (10)	−0.0054 (9)	−0.0067 (10)
O3	0.0453 (13)	0.0819 (18)	0.0500 (13)	0.0019 (12)	0.0210 (11)	−0.0035 (13)
O4	0.0533 (14)	0.0459 (15)	0.126 (2)	−0.0231 (12)	0.0493 (15)	−0.0352 (15)
O5	0.0499 (12)	0.0283 (11)	0.0514 (13)	−0.0063 (10)	0.0133 (10)	−0.0108 (10)
O1W	0.0399 (12)	0.0536 (15)	0.0716 (16)	−0.0091 (11)	0.0198 (11)	−0.0203 (13)
C1	0.0371 (17)	0.0235 (15)	0.0408 (17)	0.0012 (13)	0.0078 (14)	0.0056 (14)
C2	0.0235 (13)	0.0295 (16)	0.0369 (16)	−0.0052 (12)	0.0049 (11)	0.0030 (13)
C3	0.0399 (16)	0.0364 (18)	0.0472 (18)	0.0048 (15)	0.0013 (14)	0.0037 (15)
C4	0.056 (2)	0.069 (3)	0.047 (2)	0.0104 (19)	−0.0089 (17)	0.0112 (19)
C5	0.061 (2)	0.098 (3)	0.0355 (19)	−0.001 (2)	−0.0050 (17)	−0.007 (2)
C6	0.0492 (19)	0.060 (2)	0.051 (2)	−0.0011 (18)	0.0081 (16)	−0.0185 (19)
C7	0.0255 (14)	0.0331 (17)	0.0407 (17)	−0.0081 (12)	0.0034 (12)	0.0026 (14)
C8	0.0298 (15)	0.0320 (17)	0.0351 (15)	−0.0048 (13)	0.0086 (12)	0.0002 (14)
C9	0.0422 (18)	0.0319 (17)	0.0544 (19)	−0.0101 (14)	0.0115 (14)	−0.0043 (15)
C10	0.0339 (17)	0.051 (2)	0.066 (2)	−0.0191 (16)	0.0065 (15)	0.0003 (19)
C11	0.0255 (15)	0.064 (2)	0.060 (2)	−0.0004 (17)	0.0041 (14)	0.006 (2)
C12	0.0325 (16)	0.0381 (18)	0.0482 (18)	0.0053 (14)	0.0057 (13)	−0.0021 (15)
C13	0.0275 (15)	0.0310 (16)	0.0308 (15)	0.0000 (12)	0.0027 (11)	−0.0025 (13)
C14	0.0323 (16)	0.0254 (16)	0.0395 (16)	0.0004 (13)	0.0015 (13)	−0.0001 (14)
C15	0.070 (2)	0.075 (3)	0.070 (3)	−0.008 (2)	0.005 (2)	0.034 (2)
C16	0.0409 (18)	0.067 (2)	0.051 (2)	−0.0076 (18)	0.0062 (15)	0.0202 (19)
C17	0.059 (2)	0.092 (3)	0.039 (2)	−0.020 (2)	−0.0020 (16)	0.014 (2)
C18	0.057 (2)	0.069 (3)	0.0380 (19)	−0.0188 (19)	0.0025 (16)	−0.0017 (18)
C19	0.0316 (16)	0.050 (2)	0.0341 (16)	−0.0065 (14)	0.0096 (13)	−0.0049 (15)
C20	0.0485 (19)	0.048 (2)	0.0455 (19)	−0.0052 (16)	0.0052 (15)	0.0036 (17)
C21	0.0258 (14)	0.0451 (19)	0.0357 (17)	−0.0042 (13)	0.0090 (12)	−0.0073 (15)
C22	0.0474 (19)	0.058 (2)	0.0389 (18)	−0.0118 (17)	0.0065 (14)	−0.0176 (18)
C23	0.056 (2)	0.038 (2)	0.064 (2)	−0.0134 (16)	0.0109 (17)	−0.0206 (18)
C24	0.0435 (18)	0.0355 (19)	0.058 (2)	−0.0045 (15)	0.0119 (15)	−0.0116 (16)
C25	0.083 (3)	0.034 (2)	0.082 (3)	−0.0090 (19)	0.008 (2)	−0.003 (2)
C26	0.0398 (16)	0.0388 (19)	0.0421 (18)	−0.0015 (15)	0.0046 (13)	−0.0065 (16)

### Geometric parameters (Å, °)

**Table:**

Cu1—O5	1.915 (2)	C9—H9	0.9300
Cu1—O2^i^	1.9370 (19)	C10—C11	1.377 (5)
Cu1—N2	2.000 (2)	C10—H10	0.9300
Cu1—N1	2.018 (2)	C11—C12	1.374 (4)
Cu1—O1W	2.388 (2)	C11—H11	0.9300
N1—C20	1.336 (4)	C12—C13	1.396 (4)
N1—C19	1.349 (4)	C12—H12	0.9300
N2—C26	1.331 (4)	C13—C14	1.508 (4)
N2—C21	1.353 (4)	C15—C16	1.510 (5)
O1—C7	1.392 (3)	C15—H15A	0.9600
O1—C8	1.394 (3)	C15—H15B	0.9600
O2—C1	1.274 (3)	C15—H15C	0.9600
O2—Cu1^ii^	1.9371 (19)	C16—C20	1.384 (4)
O3—C1	1.225 (3)	C16—C17	1.388 (5)
O4—C14	1.226 (3)	C17—C18	1.371 (5)
O5—C14	1.261 (3)	C17—H17	0.9300
O1W—H1WA	0.8500	C18—C19	1.380 (4)
O1W—H1WB	0.8500	C18—H18	0.9300
C1—C2	1.512 (4)	C19—C21	1.460 (4)
C2—C3	1.383 (4)	C20—H20	0.9300
C2—C7	1.384 (4)	C21—C22	1.380 (4)
C3—C4	1.386 (4)	C22—C23	1.366 (5)
C3—H3	0.9300	C22—H22	0.9300
C4—C5	1.370 (5)	C23—C24	1.387 (5)
C4—H4	0.9300	C23—H23	0.9300
C5—C6	1.378 (5)	C24—C26	1.384 (4)
C5—H5	0.9300	C24—C25	1.494 (5)
C6—C7	1.389 (4)	C25—H25A	0.9600
C6—H6	0.9300	C25—H25B	0.9600
C8—C13	1.386 (4)	C25—H25C	0.9600
C8—C9	1.388 (4)	C26—H26	0.9300
C9—C10	1.376 (4)		
			
O5—Cu1—O2^i^	95.15 (8)	C12—C11—H11	120.2
O5—Cu1—N2	165.19 (9)	C10—C11—H11	120.2
O2^i^—Cu1—N2	93.03 (9)	C11—C12—C13	121.8 (3)
O5—Cu1—N1	90.14 (10)	C11—C12—H12	119.1
O2^i^—Cu1—N1	171.74 (10)	C13—C12—H12	119.1
N2—Cu1—N1	80.48 (10)	C8—C13—C12	117.4 (3)
O5—Cu1—O1W	96.74 (8)	C8—C13—C14	124.5 (2)
O2^i^—Cu1—O1W	93.83 (8)	C12—C13—C14	118.1 (3)
N2—Cu1—O1W	95.01 (8)	O4—C14—O5	124.8 (3)
N1—Cu1—O1W	91.83 (9)	O4—C14—C13	121.3 (3)
C20—N1—C19	119.2 (3)	O5—C14—C13	113.9 (2)
C20—N1—Cu1	126.1 (2)	C16—C15—H15A	109.5
C19—N1—Cu1	114.7 (2)	C16—C15—H15B	109.5
C26—N2—C21	119.4 (3)	H15A—C15—H15B	109.5
C26—N2—Cu1	125.53 (19)	C16—C15—H15C	109.5
C21—N2—Cu1	115.0 (2)	H15A—C15—H15C	109.5
C7—O1—C8	115.2 (2)	H15B—C15—H15C	109.5
C1—O2—Cu1^ii^	126.63 (18)	C20—C16—C17	116.2 (3)
C14—O5—Cu1	129.49 (18)	C20—C16—C15	120.8 (4)
Cu1—O1W—H1WA	99.4	C17—C16—C15	123.0 (3)
Cu1—O1W—H1WB	143.3	C18—C17—C16	120.8 (3)
H1WA—O1W—H1WB	104.5	C18—C17—H17	119.6
O3—C1—O2	126.5 (3)	C16—C17—H17	119.6
O3—C1—C2	118.6 (3)	C17—C18—C19	119.6 (3)
O2—C1—C2	114.8 (2)	C17—C18—H18	120.2
C3—C2—C7	118.6 (3)	C19—C18—H18	120.2
C3—C2—C1	118.2 (3)	N1—C19—C18	120.5 (3)
C7—C2—C1	123.2 (3)	N1—C19—C21	114.7 (2)
C2—C3—C4	121.1 (3)	C18—C19—C21	124.8 (3)
C2—C3—H3	119.5	N1—C20—C16	123.7 (3)
C4—C3—H3	119.5	N1—C20—H20	118.1
C5—C4—C3	119.5 (3)	C16—C20—H20	118.1
C5—C4—H4	120.2	N2—C21—C22	119.8 (3)
C3—C4—H4	120.2	N2—C21—C19	115.0 (3)
C4—C5—C6	120.4 (3)	C22—C21—C19	125.2 (3)
C4—C5—H5	119.8	C23—C22—C21	120.2 (3)
C6—C5—H5	119.8	C23—C22—H22	119.9
C5—C6—C7	119.8 (3)	C21—C22—H22	119.9
C5—C6—H6	120.1	C22—C23—C24	120.6 (3)
C7—C6—H6	120.1	C22—C23—H23	119.7
C2—C7—C6	120.5 (3)	C24—C23—H23	119.7
C2—C7—O1	119.6 (2)	C26—C24—C23	116.2 (3)
C6—C7—O1	119.6 (3)	C26—C24—C25	121.3 (3)
C13—C8—C9	121.2 (3)	C23—C24—C25	122.5 (3)
C13—C8—O1	121.5 (2)	C24—C25—H25A	109.5
C9—C8—O1	117.3 (3)	C24—C25—H25B	109.5
C10—C9—C8	119.8 (3)	H25A—C25—H25B	109.5
C10—C9—H9	120.1	C24—C25—H25C	109.5
C8—C9—H9	120.1	H25A—C25—H25C	109.5
C9—C10—C11	120.3 (3)	H25B—C25—H25C	109.5
C9—C10—H10	119.9	N2—C26—C24	123.8 (3)
C11—C10—H10	119.9	N2—C26—H26	118.1
C12—C11—C10	119.5 (3)	C24—C26—H26	118.1
			
O5—Cu1—N1—C20	−10.9 (3)	C10—C11—C12—C13	−0.4 (5)
N2—Cu1—N1—C20	−179.3 (3)	C9—C8—C13—C12	−0.9 (4)
O1W—Cu1—N1—C20	85.9 (3)	O1—C8—C13—C12	−179.5 (2)
O5—Cu1—N1—C19	165.91 (19)	C9—C8—C13—C14	177.8 (3)
N2—Cu1—N1—C19	−2.56 (19)	O1—C8—C13—C14	−0.8 (4)
O1W—Cu1—N1—C19	−97.33 (19)	C11—C12—C13—C8	1.0 (4)
O5—Cu1—N2—C26	131.0 (3)	C11—C12—C13—C14	−177.7 (3)
O2^i^—Cu1—N2—C26	7.5 (2)	Cu1—O5—C14—O4	−4.9 (4)
N1—Cu1—N2—C26	−177.6 (2)	Cu1—O5—C14—C13	173.43 (17)
O1W—Cu1—N2—C26	−86.6 (2)	C8—C13—C14—O4	−26.1 (4)
O5—Cu1—N2—C21	−50.3 (4)	C12—C13—C14—O4	152.6 (3)
O2^i^—Cu1—N2—C21	−173.76 (19)	C8—C13—C14—O5	155.5 (3)
N1—Cu1—N2—C21	1.10 (18)	C12—C13—C14—O5	−25.8 (4)
O1W—Cu1—N2—C21	92.12 (19)	C20—C16—C17—C18	1.6 (5)
O2^i^—Cu1—O5—C14	−76.3 (3)	C15—C16—C17—C18	179.9 (3)
N2—Cu1—O5—C14	160.5 (3)	C16—C17—C18—C19	−0.4 (5)
N1—Cu1—O5—C14	110.1 (3)	C20—N1—C19—C18	1.4 (4)
O1W—Cu1—O5—C14	18.2 (3)	Cu1—N1—C19—C18	−175.6 (2)
Cu1^ii^—O2—C1—O3	−13.0 (4)	C20—N1—C19—C21	−179.5 (3)
Cu1^ii^—O2—C1—C2	164.26 (18)	Cu1—N1—C19—C21	3.5 (3)
O3—C1—C2—C3	53.5 (4)	C17—C18—C19—N1	−1.2 (5)
O2—C1—C2—C3	−124.0 (3)	C17—C18—C19—C21	179.9 (3)
O3—C1—C2—C7	−126.4 (3)	C19—N1—C20—C16	−0.2 (5)
O2—C1—C2—C7	56.2 (4)	Cu1—N1—C20—C16	176.5 (2)
C7—C2—C3—C4	−3.1 (4)	C17—C16—C20—N1	−1.3 (5)
C1—C2—C3—C4	177.0 (3)	C15—C16—C20—N1	−179.6 (3)
C2—C3—C4—C5	1.5 (5)	C26—N2—C21—C22	−0.4 (4)
C3—C4—C5—C6	0.9 (6)	Cu1—N2—C21—C22	−179.2 (2)
C4—C5—C6—C7	−1.6 (5)	C26—N2—C21—C19	179.2 (2)
C3—C2—C7—C6	2.4 (4)	Cu1—N2—C21—C19	0.4 (3)
C1—C2—C7—C6	−177.8 (3)	N1—C19—C21—N2	−2.6 (4)
C3—C2—C7—O1	−171.6 (2)	C18—C19—C21—N2	176.4 (3)
C1—C2—C7—O1	8.3 (4)	N1—C19—C21—C22	177.0 (3)
C5—C6—C7—C2	0.0 (5)	C18—C19—C21—C22	−4.0 (5)
C5—C6—C7—O1	173.9 (3)	N2—C21—C22—C23	0.5 (4)
C8—O1—C7—C2	−125.2 (3)	C19—C21—C22—C23	−179.1 (3)
C8—O1—C7—C6	60.8 (3)	C21—C22—C23—C24	−0.4 (5)
C7—O1—C8—C13	−123.0 (3)	C22—C23—C24—C26	0.1 (5)
C7—O1—C8—C9	58.4 (3)	C22—C23—C24—C25	179.8 (3)
C13—C8—C9—C10	0.2 (4)	C21—N2—C26—C24	0.1 (4)
O1—C8—C9—C10	178.8 (3)	Cu1—N2—C26—C24	178.8 (2)
C8—C9—C10—C11	0.5 (5)	C23—C24—C26—N2	0.0 (5)
C9—C10—C11—C12	−0.4 (5)	C25—C24—C26—N2	−179.7 (3)

Symmetry codes: (i) −*x*+1, *y*−1/2, −*z*+1/2; (ii) −*x*+1, *y*+1/2, −*z*+1/2.

### Hydrogen-bond geometry (Å, °)

**Table:**

*D*—H···*A*	*D*—H	H···*A*	*D*···*A*	*D*—H···*A*
O1W—H1WA···O4	0.85	1.99	2.750 (3)	148
O1W—H1WB···O3^iii^	0.85	2.13	2.972 (3)	171

Symmetry codes: (iii) −*x*, *y*−1/2, −*z*+1/2.

## References

[bb1] Ahmadi, R., Kalateh, K. & Amani, V. (2010). *Acta Cryst.* E**66**, m562. 10.1107/S1600536810014091 PMC2979037 21579044

[bb2] Ahmadi, R., Khalighi, A., Kalateh, K., Amani, V. & Khavasi, H. R. (2008). *Acta Cryst.* E**64**, m1233. 10.1107/S1600536808027657 PMC2959444 21200993

[bb3] Brandenburg, K. (2010). *DIAMOND* Crystal Impact GbR, Bonn, Germany.

[bb4] Bruker (2009). *APEX2*, *SAINT* and *SADABS* Bruker AXS Inc., Madison, Wisconsin, USA.

[bb5] Dong, X.-Y., Xu, X. & Yang, L. (2009). *Acta Cryst.* E**65**, m1290. 10.1107/S1600536809039191 PMC2971325 21578058

[bb6] Gong, H.-Y., Bai, Y. & Liu, W. (2009). *Acta Cryst.* E**65**, m1589. 10.1107/S1600536809047679 PMC2971933 21578619

[bb7] Hong, J. (2008*a*). *Acta Cryst.* E**64**, m17. 10.1107/S1600536807062216 PMC2914909 21200518

[bb8] Hong, J. (2008*b*). *Acta Cryst.* E**64**, m21. 10.1107/S1600536807060825 PMC2914913 21200557

[bb9] Kalateh, K., Ahmadi, R., Ebadi, A., Amani, V. & Khavasi, H. R. (2008). *Acta Cryst.* E**64**, m1353–m1354. 10.1107/S160053680803119X PMC2959790 21580816

[bb10] Khalighi, A., Ahmadi, R., Amani, V. & Khavasi, H. R. (2008). *Acta Cryst.* E**64**, m1211–m1212. 10.1107/S1600536808027104 PMC2960607 21201646

[bb11] Sheldrick, G. M. (2008). *Acta Cryst.* A**64**, 112–122. 10.1107/S0108767307043930 18156677

[bb12] Wang, W., Zhang, D.-J., Fan, Y., Song, T.-Y. & Zhang, P. (2010). *Acta Cryst.* E**66**, m462. 10.1107/S1600536810010639 PMC2983807 21580547

[bb13] Xu, X., Wang, P. & Shi, S. (2008*a*). *Acta Cryst.* E**64**, m90. 10.1107/S1600536807063155 PMC2914964 21200657

[bb14] Xu, M.-L., Zhou, R., Wang, G.-Y. & Ng, S. W. (2008*b*). *Acta Cryst.* E**64**, m712–m713. 10.1107/S1600536808010805 PMC2961154 21202241

[bb15] Yaghi, O. M., Li, H., Davis, C., Richardson, D. & Groy, T. L. (1998). *Acc. Chem. Res.* **31**, 474–484.

[bb16] Yu, C.-H. (2008). *Acta Cryst* E**64**, m1106. 10.1107/S1600536808023982 PMC2960729 21201572

[bb17] Zhao, Q.-L. & Bai, H.-F. (2009). *Acta Cryst.* E**65**, m866. 10.1107/S160053680902488X PMC2977444 21583333

